# Mindfulness, mental health, and motives for eating tasty foods when not in metabolic need

**DOI:** 10.3389/fpsyg.2023.1308609

**Published:** 2024-01-19

**Authors:** Katherine G. Moore, Justess D. Rice, John E. Gampher, Mary M. Boggiano

**Affiliations:** Department of Psychology, The University of Alabama at Birmingham, Birmingham, AL, United States

**Keywords:** mindfulness-based interventions, eating behavior, non-homeostatic, psychiatric disorder, coping, hedonic, palatable, obesity

## Abstract

Habitual consumption of highly palatable foods when not in metabolic need (HPF eating) is linked to obesity. High HPF consumption is also linked to mental health disorder (MHD) symptoms. Mindfulness-based interventions are popular treatments for obesity and MHDs, but little is known about the relationship between trait mindfulness and motive-based HPF eating. Therefore, a total of 927 young adults completed a survey that included the Palatable Eating Motives Scale-7 (which identifies Coping-, Reward enhancement-, Social-, and Conformity-eating), the Mindful Attention Awareness Scale, the Perceived Stress Scale, and demographic and body mass index (BMI) questions. An MHD questionnaire allowed a comparison of HPF eating between participants with and without various MHDs. Regressions revealed that Coping-eating was independently associated with lower mindfulness and also greater perceived stress, higher BMI, and female sex. Of these variables, only lower mindfulness was independently associated with Reward-, Social-, and Conformity-eating. Coping- and Reward-eating were more frequent in participants with versus without an anxiety disorder, depression, ADD/ADHD, and PTSD. Coping-eating was also more frequent in participants with body dysmorphic disorder. These findings warrant investigations in participants with clinically validated diagnoses for DSM-specific MHDs. Results from such investigations and the uncovered nature of associations between motive-specific HPF eating and trait mindfulness could provide novel targets to improve mindfulness-based interventions for obesity and MHDs.

## 1 Introduction

Habitual eating of highly palatable foods (HPFs) for non-metabolic reasons, i.e., when not in an energy deficit (referred to here as “HPF eating”) is a key contributor to obesity (Berthoud, 2011; Johnson and Wardle, 2014). These foods—typically in the form of fast food, desserts, dessert-like drinks, and junk food—tend to be highly processed, tasty, and energy-dense due to their high sugar and fat content (Fazzino et al., 2019), rendering them easy to overeat and obesogenic. A high consumption of HPFs, which characterizes the “Western diet,” (Johnson and Wardle, 2014; Fazzino et al., 2019) has also been implicated in the development of mental health disorder (MHD) symptoms. A higher intake of HPFs is associated with a depletion of phytochemicals, vitamins, and minerals, and with an excess of trans-fats, saturated fats, high glycemic-index carbohydrates, other sugars, and calories. These nutritional changes have been found to increase inflammation, oxidative stress, and mitochondrial and HPA axis dysfunction and to suppress neurotransmitter synthesis and metabolism, neurogenesis, and healthy gut microbiota and epigenetic changes (Bremner et al., 2020; López-Taboada et al., 2020; Marx et al., 2021). In turn, these biological changes can precipitate brain and endocrine alterations to impair behavior, emotion regulation, cognition, sleep, and other functions in a manner consistent with symptoms of MHDs (Bremner et al., 2020; López-Taboada et al., 2020; Marx et al., 2021; Abdulla et al., 2023). Higher rates of some of the aforementioned biological changes have been detected in clients with MHDs compared to healthy controls (Che et al., 2010; Bauer and Teixeira, 2019). HPF eating may also worsen MHDs by compounding symptoms with the stigma and health complications associated with obesity (Emmer et al., 2020; Weiss et al., 2020). The body of evidence linking HPFs to MHDs is compelling enough to have spurred “nutritional psychiatry” as a new and growing field (Lachance and Ramsey, 2015; López-Taboada et al., 2020).

HPF eating occurs for various reasons across individuals, including individuals with obesity. One’s primary motive behind HPF eating tends to be consistent and habitual (Boggiano et al., 2015a,b). The main motives behind HPF eating include coping (Coping-eating), enhancing reward (Reward-eating), being more social (Social-eating), and conforming (Conformity-eating) (Burgess et al., 2014; White et al., 2022). HPF eating, primarily but not exclusively for coping motives, is associated with a higher body mass index (BMI) and predicts increased BMI over time (Burgess et al., 2014; Boggiano et al., 2015a; Boggiano, 2016). It is also associated with binge eating (Boggiano et al., 2014; Burgess et al., 2014; Boggiano, 2016), emotional eating, restrained eating, overconcern with body weight and shape, and with greater stress reactivity (Boggiano et al., 2017), perceived stress (Abdulla et al., 2023), HPF craving (White et al., 2022), suggestibility (Ray et al., 2020), emotional dysregulation (Orihuela et al., 2017), self-criticism, and self-judgment (Mantzios and Egan, 2018). Inarguably, these conditions are incompatible with healthy body weight and positive mental health.

Little is known about HPF eating for different motives in MHDs. Based on the ability of HPF intake to momentarily suppress physiological stress responses and negative emotional states (Tomiyama et al., 2011; Wagner and Heatherton, 2015; Mason et al., 2021), Coping-eating may be more frequent in affective (e.g., depression and anxiety) and stress/trauma-related disorders such as post-traumatic stress disorder (PTSD). Based on the reward-seeking aspects of ADD, ADHD, and substance use disorders (Davis et al., 2015; Volkow et al., 2019), Reward-eating may be more characteristic of these disorders. Based on the stress-reducing effects of HPFs and the internalization of idealized appearance in body dysmorphic disorder (BDD) (Neziroglu et al., 2008), Coping- and Conformity-eating may be more common in this disorder.

Mindfulness refers to “the awareness that emerges through paying attention on purpose, in the present moment, and non-judgmentally” (Kabat-Zinn, 2003, p.145). Mindfulness-based interventions for obesity and MHDs have surged in popularity (Rogers et al., 2017; Warren et al., 2017; Xue et al., 2019; Tran et al., 2022). However, there is a dearth of knowledge regarding the relationship between dispositional mindfulness, HPF eating, and HPF eating motives.

Mindfulness-based interventions for the treatment of obesity have generally yielded positive outcomes, but weight loss remains a challenge (Rogers et al., 2017; Warren et al., 2017). There is evidence to suggest that reducing HPF eating could help reduce body weight. For example, a pilot weight loss intervention based on attending to hunger and satiety cues (mindfulness-like awareness) found that the amount of change in Coping-, Reward-, and Social-eating predicted the amount of weight lost post-intervention (White et al., 2022). In a mindfulness-based intervention utilizing smartphones, frequency of Coping- and Reward-eating decreased alongside weight loss (Mason et al., 2018). Conversely, in a mindfulness-based intervention that resulted in no weight loss, frequency of HPF eating did not change for any of the motives (Masih et al., 2020). In non-intervention studies, young adults with coping as their main motive for HPF eating gained significantly more body weight over 2 years (Boggiano et al., 2015a). In a different study, individuals with a higher BMI gained more weight over the COVID-19 pandemic if their intake of unhealthy food was tied to coping versus not tied to coping (Mason et al., 2021). In non-clinical studies involving mindfulness, less trait mindfulness was found to be associated with a greater BMI and fat mass (Loucks et al., 2016), with disinhibited eating (Jordan et al., 2014), and with a greater reported intake of saturated fat and sugar (Mantzios et al., 2018).

Identifying one’s primary motive behind HPF eating and the extent to which the motive is related to trait mindfulness could provide behavioral targets to enhance weight loss from mindfulness-based interventions. However, it is first necessary to determine whether trait mindfulness and HPF eating are related, and if so, for what motives. In a study similar to the present one, Coping-, Reward-, Social-, and Conformity-eating were found to be associated with lower scores on the awareness facet of a trait mindfulness scale; only Coping-eating was associated with other facets of the scale (Mantzios and Egan, 2018). The present study will build on those findings by determining whether the associations generalize to a larger population and one that includes more male, Black, and Hispanic adults. It will also determine whether the associations are accounted for by other factors such as BMI—which is positively related to HPF eating (Burgess et al., 2014; Boggiano et al., 2015a; Boggiano, 2016)—and by greater perceived stress—which is related to both lower trait mindfulness (Donald et al., 2016; La Torre et al., 2022) and more frequent Coping-eating (Abdulla et al., 2023). The larger sample also allows for the testing of differences in demographic factors as possible explanatory variables.

Mindfulness-based interventions have also garnered positive results in the treatment of MHDs. Nevertheless, given the influence that diets high in HPFs can exert on the mind and behavior (Che et al., 2010; Bauer and Teixeira, 2019; Bremner et al., 2020; López-Taboada et al., 2020; Marx et al., 2021; Abdulla et al., 2023), outcomes may be improved by concomitant targeting of a client’s primary motive behind HPF eating. However, it is first necessary to investigate whether HPF eating occurs with higher frequency in individuals with versus without MHDs. To our knowledge, this has not been investigated.

Therefore, the study aimed to examine associations between trait mindfulness and the frequency of HPF eating for the various motive types while controlling for possible influencing variables and then determining the extent to which trait mindfulness explained any significant effects. The second aim was to determine the frequency of HPF eating among participants with and without an MHD diagnosis. It was intended as a preliminary investigation as it used self-diagnosis of MHDs and assessed types of MHDs in broad categories.

For the first study aim, it was hypothesized that all the HPF eating motives would be negatively associated with trait mindfulness, but that perceived stress would also be independently associated with Coping- and Conformity-eating. For the second aim, it was hypothesized that Coping-eating would be more frequent in participants with depression, an anxiety disorder, and PTSD, that Reward-eating would be more frequent in participants with ADD/ADHD and substance use disorder, and that both Coping- and Conformity-eating would be more frequent in participants with BDD. Finally, trait mindfulness was hypothesized to be lower in those with an MHD (Tran et al., 2022) such that it would account for the more frequent HPF eating in at least some of the disorders assessed.

## 2 Materials and methods

### 2.1 Participants

Participants were college students at the University of Alabama at Birmingham (UAB) who took part in a confidential and anonymous cross-sectional survey study conducted online. The consent form focused on sensory experience questions (not included here) and “other psychological measures” such that participants were blinded to the researchers’ interest in eating behavior and mindfulness. A total of 1,040 students completed the survey. Data were tested for normality with histograms, skewness, and kurtosis (Kim, 2013). Participants were omitted if they were missing data on main variables, if they were aged <18 or > 30 years (to exclude minors and obtain a sample representing a young adult population), and if they had a BMI in the underweight range (<18.5, CDC.gov, 2022). The final sample was made up of *N* = 927 adults, 74.5% female, 24.9% male, and 0.5% intersex. The mean age was 19.7 (SD = 2.11, range = 18–30). The ethnic makeup of the sample was 50.2% White, 29.7% Black, 11.7% Asian/Pacific Islander, 7.7% Hispanic or Latino, and 0.9% Other (American Indian, Alaska Native, or unspecified). The mean BMI was 26.73 kg/m^2^ (SD = 6.90, range = 18.5–67.0). Participants with BMIs ≥40 had no outlying data on the variables of interest. Students of diverse majors completed the survey as one of several other options required in Introduction to Psychology classes or for extra credit in other psychology classes. This study was approved by the UAB Institutional Review Board for Human Use #IRB-300008579.

### 2.2 Measures

#### 2.2.1 Palatable Eating Motive Scale-7

The 20-item Palatable Eating Motive Scale-7 (PEMS-7) assesses the frequency of HPF eating and drinking over the past year for four motives: Coping, Reward Enhancement, Social, and Conformity (White et al., 2022). The Coping motive measures the frequency of consuming HPFs to manage adverse emotions or situations, e.g., “I consume these foods/drinks to forget my worries”; the Reward motive refers to the pleasurable experience or sensations from the food itself, e.g., “I consume these foods/drinks because it gives me a pleasant feeling”; the Social motive refers to the increase in enjoyment with others, e.g., “I consume these foods/drinks because it helps me to enjoy a party”; and the Conformity motive refers to abiding by the pressures or demands from others, e.g., “I consume these foods/drinks because my friends or family want me to eat these foods/drinks.” The PEMS-7 (White et al., 2022) differs from the previous 19-item (Burgess et al., 2014) and 20-item PEMS (Boggiano, 2016) only in its 7- versus 5-point response scale (1 = Never to 7 = Always; higher scores indicate more frequent HPF eating for the motive) and in explicitly describing eating for “reasons other than hunger” in the instructions. The instructions provide examples of HPFs. Motive scores are the mean of the 5-item responses of the motive.

#### 2.2.2 Mindful Attention Awareness Scale

The 15-item Mindful Attention Awareness Scale (MAAS) assesses the dispositional frequency of being attentive to and aware of one’s feelings and surroundings and of being focused on the present (Brown and Ryan, 2003). An example item is, “I tend to walk quickly to get where I’m going without paying attention to what I experienced along the way.” Responses are coded 1 = Almost Always to 6 = Almost Never, and the score is the mean of the item responses such that higher scores indicate more mindfulness. This scale also has good validity for discerning MHD from non-MHD traits (Brown and Ryan, 2003; Christopher and Gilbert, 2010).

#### 2.2.3 Perceived Stress Scale

The 10-item Perceived Stress Scale (PSS) assesses dispositional frequency to appraise situations as stressful within a month (Cohen et al., 1983). Responses are coded 0 = Never to 4 = Very Often. An item example is, “Have you felt difficulties were piling up so high you could not overcome them?” The score is the sum of the 10-item responses such that higher scores indicate greater perceived stress.

#### 2.2.4 Mental health disorders questionnaire

This was an original questionnaire that listed broad categories of MHDs common among young adults (de Girolamo et al., 2012). It was brief to avoid response fatigue and was intended to provide preliminary information as to the nature of HPF eating in individuals with versus without an MHD. The list included the following: attention-deficit/attention-deficit hyperactivity disorder (ADD/ADHD), autism spectrum disorder (ASD), anxiety disorder or specific phobia (referred to here as “anxiety disorder”), body dysmorphic disorder (BDD), depression, obsessive-compulsive disorder (OCD), post-traumatic stress disorder (PTSD), and substance use disorders (including alcohol, tobacco, and nicotine). The questionnaire asked, “Have you been medically diagnosed or suspect that you might have the following condition(s)?” Response choices were “no,” “suspect,” and “diagnosed.” Participants who selected “no” and “diagnosed” made up the No Diagnosis group and the Diagnosed group, respectively, for each MHD. Participants who selected “suspect” were not included in the analyses or results for the particular MHD. The “suspect” option was included to reduce the chance that those reporting “no” (no diagnosis) may have had symptoms but had not (or had not yet) sought treatment for a formal diagnosis. It was therefore expected that some in the No Diagnosis group may have had symptoms and that some in the Diagnosed group may not currently have had symptoms, i.e., the focus was on diagnosis status, not symptoms status.

#### 2.2.5 Body mass index and demographics

Height and weight were self-reported for a BMI later calculated with the formula kg/m^2^. For analyses, BMI was used as a continuous variable but also as established categories for descriptive purposes in Table 1: healthy (BMI = 18.5–24.9), overweight (BMI = 25–29.9), and obesity (BMI ≥ 30) (CDC.gov, 2022). Age, assigned sex (0 = female, 1 = male, and 2 = intersex), and ethnicity were also obtained.

**Table 1:** Mean (SD) scores on the PEMS-7 motives, MAAS, and PSS for the entire sample and by demographic and BMI groups.

	*N*	Coping	Reward	Social	Conformity	MAAS	PSS
All	927	2.83 (1.35)	2.79 (1.25)	2.95 (1.22)	1.72 (0.82)	3.45 (0.83)	21.36 (7.04)
*Sex^a^*							
Female	691	2.98 (1.36)^***^	2.81 (1.26)	3.01 (1.23)	1.73 (0.83)	3.40 (0.82)^***^	22.35 (6.59)^***^
Male	231	2.40 (1.20)	2.73 (1.24)	2.78 (1.19)	1.68 (0.77)	3.60 (0.8)	18.21 (7.36)
*Ethnicity^b^*							
White	465	2.85 (1.31)	2.83 (1.19)	2.94 (1.23)	1.75 (0.80)	3.39 (0.80)	21.20 (7.22)
Black	275	2.70 (1.39)	2.70 (1.31)	2.88 (1.19)	1.60 (0.83)	3.51 (0.85)	21.75 (6.87)
Asian/PI	108	2.90 (1.24)	2.93 (1.32)	3.21 (1.21)	1.86 (0.80)	3.59 (0.88)	19.87 (6.34)
Hispanic	71	3.09 (1.53)	2.76 (1.36)	2.96 (1.30)	1.76 (0.85)	3.41 (0.83)	21.30 (7.34)
*BMI^c^*							
Healthy	456	2.67 (1.25)	2.75 (1.25)	2.91 (1.22)	1.71 (0.83)	3.49 (0.86)	20.92 (7.18)
Overweight	265	2.73 (1.29)	2.65 (1.18)	2.88 (1.12)	1.70 (0.81)	3.46 (0.78)	21.38 (6.78)
Obesity	206	3.32 (1.51)^***^	3.06 (1.31)^**^	3.14 (1.35)	1.76 (0.80)	3.35 (0.80)	22.33 (7.0)

PEMS-7, Palatable Eating Motives Scale-7; BMI, body mass index; MAAS, Mindful Attention Awareness Scale; PSS, Perceived Stress Scale. ^a^Does not include intersex due to small sample size (*N* = 5). ^b^Does not include “Other” ethnicity due to small sample size (*N* = 8). ^c^BMI *post-hoc* comparisons for Coping: Obesity > Overweight = Healthy (*p* < 0.001); for Reward: Obesity > Overweight (*p* < 0.001) and Obesity > Healthy (*p* < 0.01); ^**^p < 0.01, ^***^p < 0.001 main effect difference between the groups.

### 2.3 Procedures

Participants accessed the survey via an electronic link. The survey took approximately 20–30 min to complete and opened with a statement asking that they complete the survey in private and when they were not in a rush.

### 2.4 Statistical analyses

Pearson’s r determined correlations between PEMS-7 motives and MAAS scores; partial r controlled for BMI and PSS scores, separately. Linear regressions with MAAS, PSS, BMI, and demographics as independent variables and the HPF eating motives as dependent variables allowed a comparison of the strength of correlations between variables per motive and across motives. Separate ANOVAs with Bonferroni *post-hoc* tests determined differences between demographic and BMI groups on the measures. ANCOVAs assessed the effect of MHD status on HPF eating motives covarying separately for MAAS scores and BMI. Only differences with an alpha level <0.05 and a partial eta-squared *ƞ*^2^_p_ ≥ 0.01 effect size were denoted as significant (ƞ^2^_p_ cutoffs: 0.01 = small, 0.06 = medium, 0.14 = large) (Cohen, 1988). Data are reported as means and standard deviations (SD) except where noted. SPSS v. 28 was used to analyze the data.

## 3 Results

### 3.1 Sample scores on measures

Mean PEMS-7 motives, MAAS, and PSS scores are provided in Table 1. These are listed for the entire sample and by demographic and BMI categories. Overall, the frequency of Conformity-eating was numerically the least, and Social-eating was the most frequent motive behind HPF eating. Women had more frequent Coping-eating, lower trait mindfulness, and greater perceived stress than men. Ethnic groups did not differ on any of the measures. Finally, participants with a BMI in the obesity range had more frequent Coping- and Reward-eating than those in the overweight and healthy BMI range. BMI groups did not differ in the level of trait mindfulness or perceived stress. Age, not included in Table 1 because it is a continuous variable, was correlated with Reward-eating (*r* = 0.082, *p* < 0.05); older participants ate more frequently for this motive than younger ones. Age was uncorrelated with mindfulness and perceived stress levels.

### 3.2 Associations between HPF eating motives and trait mindfulness

As shown in Table 2, a greater frequency of HPF eating for all the PEMS-7 motives was associated with lower trait mindfulness. Associations were strongest for Coping-eating and weakest for Social-eating. Also evident in Table 2 is that the associations remained significant when controlling for differences in BMI and perceived stress. Of these two variables, greater perceived stress accounted for more of the negative association between HPF eating and trait mindfulness. The significant differences denoted in Table 1 suggested BMI, and assigned sex for Coping-eating, as possible explanatory factors for HPF eating. These variables together with perceived stress and mindfulness scores were tested as independent correlates of HPF eating in the regression analyses. As shown in Table 3, linear regressions revealed that a higher BMI, greater perceived stress, lower mindfulness, and female-assigned sex were independent explanatory variables for Coping-eating. Of these variables, only lower mindfulness was significant for Reward-, Social-, and Conformity-eating. Age and dummy-coded ethnicity were tested as independent variables but were not significant. The models accounted for a very small variance in motive-based HPF eating with R^2^ ranging from 0.05 to 0.20. However, the regressions were conducted not to find the best models of HPF eating but to assess the association of mindfulness and HPF eating unrelated to BMI and perceived stress. An inspection of the standardized betas (β) in Table 3 for Coping-eating indicated that perceived stress was more strongly associated with this type of HPF eating than lower mindfulness. A review of the unstandardized betas (*B*) indicated that Reward-eating was the type of HPF eating most strongly associated with lower mindfulness.

**Table 2:** Bivariate and partial correlations between PEMS-7 motives and MAAS scores controlling for BMI and PSS scores.

PEMS-7 motives	MAAS	MAAS partial *r* (controlling for BMI)	MAAS partial *r* (controlling for PSS)
Coping	−0.304^***^	−0.297^***^	−0.195^***^
Reward	−0.232^***^	−0.228^***^	−0.196^***^
Social	−0.197^***^	−0.193^***^	−0.164^***^
Conformity	−0.205^***^	−0.202^***^	−0.179^***^

PEMS-7, Palatable Eating Motives Scale-7; MAAS, Mindful Attention Awareness Scale; PSS, Perceived Stress Scale. ^***^p < 0.001 with a 99% confidence interval.

**Table 3:** Linear regressions with measures (BMI, PSS scores, and MAAS scores) as explanatory independent variables (IVs) and HPF eating for each PEMS-7 motive as the dependent variable (DV).

DV	IVs	*B*	SE	*β*	*t*	*p*
Coping	Constant	2.07				
	BMI	0.028	0.006	0.146	4.90	<0.001^***^
	PSS	0.052	0.006	0.272	8.29	<0.001^***^
	MAAS	−0.299	0.052	−0.185	−5.80	<0.001^***^
	Sex^a,b^	−0.284	0.095	−0.092	−3.00	0.003^**^
Reward	Constant	2.55				
	BMI	0.009	0.006	0.049	1.53	0.127
	PSS	0.010	0.006	0.057	1.66	0.098
	MAAS	−0.319	0.052	−0.211	−6.12	<0.001^***^
	Age^b^	0.045	0.019	0.075	2.33	0.020^*^
Social	Constant	3.45				
	BMI	0.007	0.006	0.039	1.29	0.227
	PSS	0.010	0.006	0.056	1.61	0.109
	MAAS	−0.256	0.051	−0.173	−4.99	<0.001^***^
Conformity	Constant	2.20				
	BMI	0.002	0.004	0.014	0.447	0.655
	PSS	0.006	0.004	0.048	1.38	0.167
	MAAS	−0.188	0.034	−0.191	−5.51	<0.001^***^

MAAS, Mindful Attention Awareness Scale; PSS, Perceived Stress Scale; PEMS-7, Palatable Eating Motives Scale-7. ^a^Does not include *N* = 5 intersex. ^b^Included because sex and age differed for these motives (Table 1). Constant *B* = mean motive score when IVs are 0. ^***^p < 0.001, ^**^p < 0.01, ^*^p < 0.05.

### 3.3 Effect of MHD status on HPF eating

**Figure 1:**
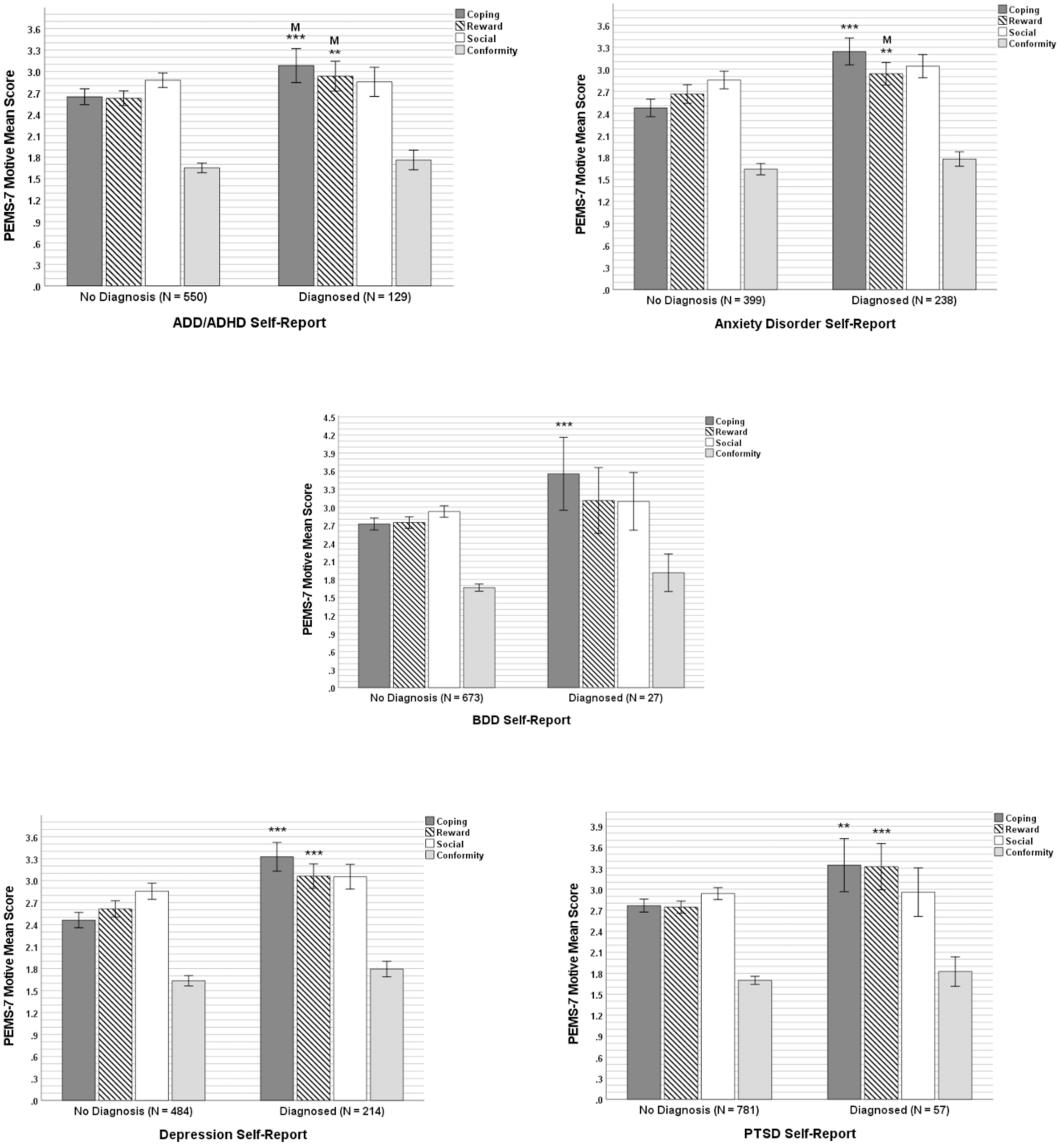
Effect of reported mental health disorder status (No Diagnosis versus Diagnosed) on the frequency of HPF eating for each of the four PEMS-7 motives for ADD/ADHD, anxiety disorder, BDD, depression, and PTSD. Error bars are 95% CI; ^*^p < 0.05, ^**^p ≤ 0.01, ^***^p ≤ 0.001 versus corresponding “No” group. “M” over the asterisks indicates that the difference was no longer significant when mindfulness (MAAS scores) was covaried. *η*^2^_p_ for ADD/ADHD: Coping = 0.016, Reward = 0.010; anxiety disorder: Coping = 0.076, Reward = 0.011; BDD: Coping = 0.014; depression: Coping = 0.090, Reward = 0.027; and PTSD: Coping = 0.012, Reward = 0.014.

As depicted in Figure 1, the frequency of HPF eating was overall higher in participants with versus without an MHD. The greater frequency of HPF eating in the Diagnosed group was most commonly Coping-eating, followed by Reward-eating, then Conformity-eating. As noted in Figure 1, Social-eating did not differ between the Diagnosed and No Diagnosis groups across MHDs. There was no difference in HPF eating between the Diagnosed and No Diagnosis groups for OCD or substance use disorder (not graphed). For ASD, the lower frequency of Social-eating in the Diagnosed versus No Diagnosis groups was <0.05 (*p* = 0.008) but *ƞ*^2^_p_ was <0.01, so the difference was not denoted as significant. For OCD, Coping-eating was higher in the Diagnosed versus No Diagnosis groups (*p* = 0.011), but *ƞ*^2^_p_ was also <0.01.

### 3.4 Effect of MHD status on trait mindfulness

As shown in Table 4, levels of trait mindfulness were lower in the Diagnosed group than in its respective No Diagnosis group for most of the MHDs.

**Table 4:** Percentage of sample and mean MAAS scores of participants reporting an MHD (Diagnosed group) and not having the MHD (No Diagnosis group).

	No Diagnosis group		Diagnosed group		
MHD	*N*%	MAAS mean (SD)	*N*%	MAAS mean (SD)	*p*
ADD/ADHD	59.8	3.68 (0.78)	13.9	3.02 (0.79)	<0.001^***^
ASD	89.3	3.51 (0.81)	1.41	3.40 (0.88)	0.606
Depression	52.5	3.67 (0.84)	23.1	3.15 (0.71)	<0.001^***^
Anxiety	43.3	3.66 (0.83)	25.7	3.13 (0.74)	<0.001^***^
PTSD	84.4	3.50 (0.83)	6.2	3.16 (0.75)	0.006^**^
BDD	73.1	3.55 (0.84)	2.9	3.03 (0.68)	0.003^**^
SUD	91.6	3.47 (0.82)	1.94	3.01 (0.73)	0.053
OCD	91.6	3.50 (0.83)	4.4	3.17 (0.74)	0.037^*^

MHD, mental health disorder; % out of a total of 927 participants; MAAS, Mindful Attention Awareness Scale; ADD/ADHD, attention-deficit disorder/attention-deficit hyperactivity disorder; ASD, autism spectrum disorder; PTSD, post-traumatic stress disorder; BDD, body dysmorphic disorder; SUD, substance use disorder; OCD, obsessive-compulsive disorder. ^**^p < 0.01, ^***^p < 0.001 difference between MAAS scores between the groups.

### 3.5 Effect of covarying mindfulness and BMI on differences in HPF eating between MHD Diagnosed and No Diagnosis groups

Given the significant differences in mindfulness between diagnosis groups (Table 4), ANOVAs were re-conducted, this time covarying for MAAS scores. Variance in trait mindfulness levels accounted for the greater frequency of HPF eating between the Diagnosed and No Diagnosis groups for Coping-eating in ADD/ADHD and for Reward-eating in ADD/ADHD and anxiety disorder; that is, the Diagnosed and respective No Diagnosis groups no longer differed when covarying for mindfulness scores. This is denoted in Figure 1 with an “M” over the bar. Considering the higher incidence of obesity in MHDs (Simon et al., 2006; Taylor et al., 2012; Weiss et al., 2020), any possible confounding association between BMI and an MHD diagnosis was tested by covarying BMI. Doing so had no effect on the significant differences between the Diagnosis and No Diagnosis groups asterisked in Figure 1.

## 4 Discussion

This study investigated the relationship between motives for consuming tasty foods and drinks when not in metabolic need (HPF eating) and trait mindfulness in a large sample of college students. A preliminary test also investigated, in the same participants, the occurrence of motive-based HPF eating between participants with versus without an MHD.

### 4.1 Associations between mindfulness and HPF eating controlling for demographics, BMI, and perceived stress

As hypothesized, greater frequency of HPF eating for all the motives was associated with lower trait mindfulness. Not predicted was that women would have a lower mean level of trait mindfulness than men. This could in part explain the higher frequency of HPF eating in women versus men found here and in previous studies (Burgess et al., 2014; Boggiano, 2016; Boggiano et al., 2017). Considering the significant contributing role of HPF eating in obesity (Johnson and Wardle, 2014; Fazzino et al., 2019), the negative associations between all the motive types of HPF eating and trait mindfulness strengthen the rationale for mindfulness-based interventions to treat obesity (Johnson and Wardle, 2014; Fazzino et al., 2019). They also give confidence that the same associations found by Mantzios and Egan (2018) with a different mindfulness subscale are not likely to be explained by any differences in perceived stress, BMI, or demographics of the participants. The subscale in that study was the “Acting with Awareness” facet of the Five Facet Mindfulness Questionnaire—Short Form (FFMQ-SF) (Mantzios and Egan, 2018). This is the subscale most associated with components of mindfulness measured by the MAAS (Zhuang et al., 2017). In the present study, Coping-eating stood out from the other motive types of HPF eating in its independent association not only with lower mindfulness but with female sex, a greater BMI, and greater perceived stress. An association with these other variables, together with a β value that was larger for perceived stress than for mindfulness, suggests that more than mindfulness techniques may be needed to reduce this type of HPF eating to affect weight loss, especially in women.

### 4.2 HPF eating motives in participants with and without an MHD diagnosis

As hypothesized, HPF eating was more frequent in the Diagnosed versus No Diagnosis groups for the majority of MHDs examined. Here, HPF eating for coping also stood out from the other motives in that it characterized most of the higher frequency of HPF eating in the MHDs examined. Reward enhancement was also a common motive in the higher frequency of HPF eating. In sharp contrast, the frequency of HPF eating for social motives did not differ between the Diagnosed and No Diagnosis groups (or between BMI groups), strengthening the status of this motive as the least associated with health consequences (Burgess et al., 2014; Boggiano, 2016; Boggiano et al., 2017). Coping and reward motives differ conceptually from social and conformity motives in that they are driven by internal factors (one’s own feelings and sensations) versus external factors. This difference may help explain why Coping- and Reward-eating were most strongly associated with lower mindfulness. It may also provide a mechanistic clue for the greater frequency of Coping- and Reward-eating in participants with obesity and MHDs. An advantage of mindfulness skills is that they help regulate responses to both internal and external stimuli (Brown and Ryan, 2003).

Coping- but not Conformity-eating was more frequent in those with BDD. That this finding was observed with only *N* = 27 individuals with BDD is noteworthy and increases confidence that results will replicate when using clinically validated assessments of this disorder. Assessment with a greater number of individuals with BDD may find Conformity-eating to also be more frequent because, in this study, Conformity-eating was numerally but not significantly more frequent in the BDD Diagnosed group. For substance use disorder, the small number of participants with this diagnosis, *N* = 18, may have confounded the observation of a difference in HPF eating relative to participants without this disorder. An investigation of HPF eating in a greater number of individuals with substance use disorders and its subtypes is worth conducting and may help elucidate why some with the disorder develop obesity while others do not (Sansone and Sansone, 2013). For participants with ASD, it is noteworthy that there was a statistical trend for HPF eating to differ from the No Diagnosis group (*p* = 0.008; *ƞ*^2^*_p_* < 0.01) despite only *N* = 13 in the Diagnosed group. Interestingly, the trend was for Social-eating to be lower, not higher; however, this trend is rational considering the social difficulties that characterize ASD. The unique lower frequency also increases confidence in the integrity of self-reporting on the MHD questionnaire. For OCD, clinical assessment versus self-report of this MHD is predicted to reveal more frequent Coping-eating compared to individuals without OCD. This is because the observed value of *p* for a difference was <0.05. However, we deemed the effect size to be too low to report as significant. If future studies find Coping-eating to be more frequent in clients with OCD, reducing it as part of the treatment protocol may help maintain success in breaking the obsessive-compulsive cycle given the similar repetitive and compulsive nature of HPF eating.

### 4.3 Limitations

Self-reported BMI and diagnosis of MHD were limitations of this study. Measured BMI and the use of clinically validated scales and/or testing of client populations will help validate the results; therefore, the MHD results should be regarded as preliminary. The MHD questionnaire was limited and did not include eating disorders. Specific classes of substance use disorders should also be examined. Self-report of HPF eating was also a limitation, but one tempered by the ecological and predictive validity of the PEMS-7 (Boggiano et al., 2015a,b; Sylvester et al., 2019; White et al., 2022). The results obtained in the college sample may not generalize to younger, older, or same-aged individuals experiencing poverty. Finally, more may be learned by using mindfulness scales with measures that include mindfulness constructs other than attentive awareness, such as the FFMQ-SF (Mantzios and Egan, 2018).

### 4.4 Strengths

The young adult sample assessed was large and had a wide BMI range and a mean BMI very similar to the national mean BMI of 27.5 for adults aged 18–25 (Ellison-Barnes et al., 2021). Though not measured, self-reported BMI tends to correlate highly with measured BMI and waist circumference (Okamoto et al., 2017). The sample was also ethnically diverse and, apart from just a higher percentage of Black people and Asian/PI people and just a lower percentage of Hispanic people, the ethnic makeup reflected that of the US population (Census.gov, 2022). Furthermore, to the best of our knowledge, this was the first study to examine relationships between mindfulness and HPF eating for various motives while controlling for potentially related factors and the first study to explore HPF eating motives in MHDs. Although diagnoses were self-reported, there is evidence of a high correlation between self-reported and clinically-diagnosed psychopathology (Sordo Vieira et al., 2022). In addition, the incidence of ADD/ADHD, anxiety disorder, depression, OCD, and PTSD parallels rates reported by the American College Health Association’s National College Health Assessment in Spring 2022 on *N* = 54,204 students (ACHA.org, 2022). The “yes/no” response method also parallels the method used by the ACHA. A strength over the dichotomous “yes/no” response method was the inclusion of a “suspect” response option to help decrease the chance that the No Diagnosis groups included participants who may have had symptoms but had not (yet) sought treatment. Finally, the results should spur studies aimed at uncovering key factors underlying the connection between HPF eating and mindfulness. Emotional dysregulation may be one factor. Mindfulness practices improve emotional regulation (Kabat-Zinn, 2003; Roemer et al., 2015) and, in adolescents, emotional dysregulation mediated the relationship between high BMI and Coping-eating (Orihuela et al., 2017). Disparities in emotional regulation may also explain why HPF eating was found to be more frequent in participants with versus without an MHD. It may also explain why mindfulness was most strongly associated with Coping- and Reward-eating motives which are motives driven by internal factors, e.g., emotions.

## 5 Conclusion

Increasing attentive awareness is a central goal of mindfulness training (Kabat-Zinn, 2003). Instruments such as the PEMS-7 can raise awareness of one’s primary motive to eat when not in caloric deficit by identifying the motive. Such instruments, together with the associations found between trait mindfulness and HPF eating motives, and the studies we hope will be inspired by the preliminary MHD findings, have the potential to improve mindfulness-based interventions for obesity and psychopathology.

## Data availability statement

The raw data supporting the conclusions of this article will be made available by the authors, without undue reservation.

## Ethics statement

The studies involving humans were approved by the UAB Institutional Review Board for Human Use #IRB-300008579. The studies were conducted in accordance with the local legislation and institutional requirements. The participants provided their online checked informed consent to participate in this study.

## Author contributions

KM: Conceptualization, Data curation, Formal analysis, Funding acquisition, Investigation, Methodology, Visualization, Writing – original draft, Writing – review & editing. JR: Data curation, Funding acquisition, Investigation, Writing – review & editing. JG: Data curation, Resources, Software, Writing – review & editing. MB: Conceptualization, Data curation, Formal analysis, Funding acquisition, Methodology, Supervision, Visualization, Writing – original draft, Writing – review & editing.
